# Absence and Presence of Human Interaction: The Relationship Between Loneliness and Empathy

**DOI:** 10.3389/fpsyg.2020.00768

**Published:** 2020-05-19

**Authors:** Tingyun Hu, Xi Zheng, Miner Huang

**Affiliations:** Department of Psychology, Sun Yat-sen University, Guangzhou, China

**Keywords:** loneliness, empathy, social support, choice, emotion regulation

## Abstract

Loneliness is the negative experience of a discrepancy between the desired and actual personal network of relationships. Whereas past work have focused on the effect of loneliness on prosocial behaviors, the present research addressed the gap by exploring the effect of loneliness on empathy. Empathy is the emotional reaction of sharing in others’ internal experiences. We adopted a new paradigm-empathy selection task, which uses free choices to assess the desire to empathize. Participants made a series of binary choices, selecting situations that instructed them to empathize or objectively describe. Results from two studies showed that, compared to non-lonely people, lonely people were more likely to choose positive empathy but to avoid negative empathy. The pattern occurs because lonely people perceived higher (vs. lower) social support in the positive (vs. negative) empathy tasks. Moreover, empathy served to be an adaptive emotion regulation strategy developed by lonely people to reduce their loneliness effectively. This research has resulted in both theoretical contributions to prosocial behavior literature and the further discovery of practical implications for loneliness intervention.

## Introduction

Despite the popularity of mobile communication and social networks, people are more socially isolated than ever before (Cacioppo et al., 2015). Loneliness refers to “a subjective distressing emotion results from a discrepancy between one’s actual and desired social relationships” (Cacioppo and Patrick, 2008). Numerous studies have demonstrated the risks of loneliness on physical health and psychological well-being. Loneliness has been shown to predict increased morbidity and mortality (reviewed in Hawkley and Cacioppo, 2010), such as increased vascular resistance (Cacioppo et al., 2002), elevated blood pressure (Hawkley et al., 2006), a higher risk of obesity (Lauder et al., 2006), and increased likelihood of engagement with unhealthy behaviors (Nieminen et al., 2013). Also, numerical studies have suggested that loneliness is associated with a poor mental health outcome (reviewed in Heinrich and Gullone, 2006), including depression (Cacioppo et al., 2010; Teo et al., 2013), decreased life satisfaction (Salimi, 2011), compulsive Internet use behaviors (Kim et al., 2009), and deliberate self-harm (Rönkä et al., 2013).

Accumulating evidence has indicated that loneliness is negatively correlated with prosocial behaviors. People who are lonely act less prosocially than others (Cassidy and Asher, 1992; Gest et al., 2001; Woodhouse et al., 2011). Besides, having one stable social relationship (e.g., a marriage) is proved to promote adults’ prosocial acts (Dye, 1980). In contrast, other research has demonstrated that the need to belong motivates lonely people to be more prosocial when they are asked to help in public situations (Huang et al., 2016) or when the targets are similarly alienated (Lee and Park, 2019). Yet, the relationship between absence (loneliness) and presence (empathy) of human connections has not been thoroughly investigated. The goal of this research was to advance our understanding of loneliness. Specifically, we examined whether loneliness would alter the course of empathic episodes.

### The Effect of Loneliness on Social Functioning

Before turning to this question, we reviewed studies in which loneliness has been shown to affect individuals’ social functions. Loneliness is a subjective feeling, and it is therefore not directly related to the actual number and frequency of social interactions. What really matters is the quality of the actual social relationships. Besides, loneliness is different from solitude (Burger, 1995). People can feel lonely when they are in a crowd or not feel lonely when they are alone. A sense of passive social isolation can leave people lacking a sense of social inclusion and belonging, which is unpleasant and undesirable.

Loneliness is such a painful experience that people will do practically everything to avoid it. There are two conflicting perspectives on how people respond when they are suffering from loneliness, which are loneliness-perpetuation and loneliness-reduction (Vanhalst et al., 2012). The loneliness-perpetuation perspective posits the detrimental effects of loneliness on social functioning (e.g., Anderson and Martin, 1995). Lonely people are more likely to cope with stressors by withdrawing rather than by active coping (Heinrich and Gullone, 2006; Cacioppo and Patrick, 2008). When people feel lonely, they tend to be shyer, more anxious, more socially awkward, and have lower self-esteem (Cacioppo et al., 2006). Loneliness is also associated with an implicit hypervigilance to social threats and a tendency to act toward others in a less trusting and more hostile fashion (Duck et al., 1994; Anderson and Martin, 1995; Cacioppo and Patrick, 2008), which has a cascading effect on social cognition (Twenge et al., 2003).

In contrast, a loneliness-reduction perspective takes an evolutionary approach, positing that a feeling of loneliness is adaptive in that it motivates humans to pursue and maintain social connections, thereby enhancing the survival of oneself and one’s offspring (Boomsma et al., 2005). Feeling socially isolated, lonely individuals are more socially anxious and have a stronger desire for social connection (Segrin and Kinney, 1995). Numerous studies have supported the idea that loneliness adaptively influences sensitivity to social information. For example, lonely people are more likely to remember collective and interpersonal social events and to mimic others’ behaviors to form social connections (Gardner et al., 2005). Also, loneliness predicts higher attention to emotional vocal tone (Pickett et al., 2004). Using functional magnetic resonance imaging, Cacioppo et al. (2009) found that unpleasant loneliness predicted greater activation of the visual cortex to pictures of people than of objects. Additionally, feelings of socially isolation had an influence on their behaviors. Wang et al. (2012) proved that lonely people are more willing to purchase minority-endorsed products, but they will shift preference to majority-endorsed products in public contexts. The lonely individuals also tend to seek physical warmth to ameliorate social coldness (Shalev and Bargh, 2015).

From the above, loneliness increases the motivation for searching for social interaction and reconnection. However, lonely people tend to anticipate rejection and thus tend to engage in self-protective behaviors, which results in a deficit in their social skills. Therefore, it can be predicted that the reason behind lonely people reducing their prosocial behaviors lies in their motivation for avoiding social disapproval and a negative emotional outcome (e.g., DeWall et al., 2009). But, in cases when prosocial behaviors are accompanied by social rewards (Williamson and Clark, 1989), lonely people will be motivated to act more prosocially. In the present research, we have speculated that empathy, which is considered as a relatively less risky and socially rewarding prosocial act (Batson, 1987), would be motivated by loneliness.

### Contextual Factors Affecting Empathy

*Empathy* is defined as the ability and propensity to share in and understand others’ experiences vicariously (Decety and Cowell, 2014). It can also be described as an emotional reaction that stems from another’s emotional state and is congruent with that state (Batson, 2009). Feeling empathy for the person in need is the best-documented source of altruistic motivation (Batson, 1987). More importantly, empathy performs its social function by allowing people to approach affiliation and to enhance ingroup identification (Zaki, 2014). Thus, empathy plays an essential role in interpersonal relations, including early attachment between a primary caregiver and child (Decety et al., 2012), caring for the well-being of others (Batson, 2012; Keysers and Gazzola, 2014) and facilitating cooperation, helping, and beneficial interactions among group members (Preston, 2013).

Given the important role of empathy for social well-being and prosocial behaviors, numerous studies have identified factors that influence empathy. Empathy is indicated to be moderated by how the target is perceived, including how likeable the target is to the observer (Cheng et al., 2010), the group membership of the target (Montalan et al., 2012), and the implicit attitudes of the observer (Decety et al., 2009). People avoid empathy-eliciting stimulations when empathy costs materials or time (Pancer et al., 1979; Shaw et al., 1994) and when it entails vicarious psychological costs, such as guilt (Andreoni et al., 2017), and when being emotionally overwhelming and exhausted (Cameron and Payne, 2011; Cameron et al., 2016). The evidence has suggested that, although empathy exhibits features of automaticity, empathy processes are deeply context dependent and less automatic than psychologist have expected. When deciding whether to choose empathy, people may weigh the expected value of mental or material costs along with offsetting rewards. Here, we have highlighted two contextual features (i.e., subjective cognitive cost and need for social support) that shift empathy.

It has been well-documented that empathy can be overly costly (Zaki, 2014). Except for the material costs or emotional costs that empathy may entails, the latest research (Cameron et al., 2019) found that people robustly feel that empathy is cognitively taxing, rating it as more effortful, aversive, and inefficacious than comparison tasks. Cognitive costs of empathy may derive in part from uncertainty about others’ experience and the risk of making errors (Dunn et al., 2017), and this may directly cause empathy avoidance. Research indicated that by experimentally increasing perceived efficacy of empathy (e.g., shortening the time required for empathy and providing positive feedbacks after completing each empathy task), subjective cognitive cost would be decreased, and, consequently, empathy avoidance would be eliminated (Cameron et al., 2019).

Even though resonating with others is always mentally demanding and sometimes emotionally overwhelming, an individual would voluntarily subject him/herself to its cost under certain circumstances. Although paradoxical from a hedonic perspective, such behavior becomes sensible when considered in the broader scheme of human motivation (Higgins and Pittman, 2008) because people commonly report the maintenance of strong personal relationships as a primary life goal, and they attach much importance to supportive interactions among people (Gable, 2006). Social support is defined as information leading the subject to believe that he/she is cared for and loved, esteemed, and a member of a network of mutual obligations (Cobb, 1976). It was noted that, driven by the need for social support, people may engage more deeply with others’ internal states, and empathizing with strangers can even create vicarious perception of social support and lead to a feeling of reconnection to social networks (Zaki, 2014).

Taken together, we have proposed that when the need for social support is strong enough, the negative impact of cognitive cost will be offset, and thus people will be motivated to share in others’ emotional experience, especially under the circumstances of empathizing with positive emotions. Furthermore, based on the previous theoretical and empirical research on the effect of loneliness on social functioning, it can be proposed that subjective loneliness is a critical inducement to stimulate the desire for social support, and it is, therefore, indicated to be certain motivator for positive empathy and a hindrance for negative empathy. Few studies have shed light on our hypotheses. Psychological social stress is demonstrated to influence human processing of emotional stimuli and enhance the accurate recognition of emotions, such as happiness, surprise, and anger (Barel and Cohen, 2018). Recently, evidence from an eye-tracking study (Saito et al., 2020) suggested that warm (vs. competitive) human faces automatically captured more attention from lonely individuals.

### The Beneficial Effect of Empathy on Loneliness Intervention

The latest review summarized that the variety of effective coping strategies can be grouped into six dimensions, including enhancing perceived social support (Rokach, 2018). But, so far, interventions that enhance social support have been limited to allowing lonely people to take animal-assisted therapy (Banks et al., 2008) or participate in community activities (Collins and Benedict, 2006; Stewart et al., 2009). Previously, we predicted that the desire for social support would motivate lonely people to engage more in positive empathy. Additionally, existing research has demonstrated that one’s ability to empathize has been shown to be inversely correlated with loneliness across the adult lifespan (Beadle et al., 2012). Studies in the field of organizational psychology (Soler et al., 2017) also suggested that empathy plays an important role in the increase of the occupational well-being and probably helps to prevent loneliness for healthcare professionals. Thus, we have predicted that, during the process of positive empathy, lonely people can obtain social support and thereby reduce their perceived loneliness.

### Study Overview

This research presents two experiments to investigate the effect of loneliness on empathy engagement and the potential mechanism underlying this effect. In Experiment 1, we examined whether lonely individuals would be more willing to empathize with strangers than non-lonely individuals (H_1_), and we also examined whether the relationship between loneliness and empathy tendency would be mediated by perceived social support but not subjective cognitive cost (H_2_). Moreover, we examined the role of empathy in decreasing perceived loneliness (H_3_). With a similar procedure, Experiment 2 introduced a distinction regarding the valence of empathy, namely positive vs. negative empathy, and investigated the interaction effect of the valence of empathy on the manipulation of loneliness on the desire to empathize as well as on loneliness intervention (H_4_).

## Experiment 1

Instead of a self-report measurement, we adopted a new paradigm – the empathy selection task – which uses behaviorally revealed preferences to measure motivated empathy engagement (Cameron et al., 2019). The rationale behind the measurement lies in that people develop an emotional regulation strategy whereby people choose situations to enter into based on the emotions they want to feel (Gross and Thompson, 2007). For example, people maintain distance from an empathic stimulation to avoid generating guilt (Andreoni et al., 2017). In the empathy-selection task, participants were presented with a photo of a person, and were asked to choose between two card decks over repeated measures. If they chose the empathy deck, they would be instructed to share in the experiences of the person and indicate the person’s internal experiences, and, if they chose the objective deck, they would be instructed to remain detached and indicate external features. Our dependent variable was the proportion of choosing the empathy task across trials and post-task assessments of the empathy decks. Experiment 1 primarily focused on the effect of loneliness on positive empathy engagement tendency and examined the mediating role of perceived social support and subjective cognitive cost. If feelings of being isolated would facilitate people to empathize with strangers, lonely (vs. non-lonely) people would perceive higher level of social support and be engaged in more empathy tasks.

### Methods

#### Participants

Ninety-five college students (48 males, 47 females, *M*_age_ = 20.21 years, *SD*_age_ = 2.03) were recruited to participate in this experiment. They were informed that they were making a contribution to a face database by offering their feedback toward specific facial expressions. Participants received financial rewards after completing the experiment, and all of them provided written informed consent according to the study protocol, which was approved by the Ethics Committee of the Department of Psychology, Sun Yat-sen University.

#### Design and Procedure

Participants were randomly assigned to a lonely or control recall condition and instructed to recall and spend a few minutes writing about a time they felt lonely and isolated (lonely) or their daily routine (control). After the written task was performed, their subjective feeling of loneliness was captured on the revised version of the self-report UCLA loneliness scale designed by Russell et al. (1980). The revised version of the UCLA loneliness scale was adopted for the reason that the revised version counters the possible effects of response bias in the original scale and reports concurrent reliability (α = 0.94) and validity. Besides, for the purpose of assessing participants’ perceived loneliness before and after the empathy-selection task in the present research, the loneliness scale was divided into two subscales. A total of 10 items were selected for measurement in time 1, including five of the positively worded items (e.g., “I feel in tune with the people around me”) and five of the negatively worded items (e.g., “My interests and ideas are not shared by those around me”). The remaining 10 items of the revised UCLA loneliness scale (e.g., “I have a lot in common with the people around me”; “There is no one I can turn to”) formed the other subscale to assess their post-task loneliness. The positively worded items were reversed before scoring, and hence high scores would reflect feelings of loneliness. The subscale in Time 1 achieved good levels of reliability (α = 0.90), as did the subscale in Time 2 (α = 0.88).

After completing the assessment of pre-task loneliness, participants were instructed to complete 30 trials of the empathy selection task. In each trial, participants were shown an image of a person and were instructed to choose between the decks freely. The face stimuli were taken from the CAS-PEAL Large-Scale Chinese Face Database (Gao et al., 2008). We used 15 male and 15 female models in Experiment 1, and the faces were converted to gray-level images. Each face model was depicted as experiencing positive states and presented against a white background (Supplementary Presentation S1). The objective deck was always on the left, labeled “Describe,” and the empathy deck was always on the right, labeled “Feel.” If participants chose the objective deck, they were instructed, “Look at the person in the picture, and try to notice the details of this person. Please write a sentence describing the age and gender of this person.” If participants chose the empathy deck, they were instructed “Look at the person in the picture, and try to feel what this person is feeling. Empathically focus on the internal experiences and feelings of this person. Please write one sentence describing the experiences and feelings of this person.” Trials were randomized. Participants could submit answers until 10 s had passed. After the empathy-selection task, participants were instructed to finish the subscale to assess their post-task loneliness. Finally, participants were asked to complete the assessment of the empathy decks on seven-point scales (anchored: 1 = strongly disagree, 7 = strongly agree). Participants were asked to rate the degree to which they agreed with four items adapted from MSPSS-C (Chou, 2000) as an assessment of perceived social support (e.g., “During most of the empathy decks, I feel like I was sharing joys and sorrows with the person”; “Most of individuals in the empathy decks were a source of comfort to me”; α = 0.89). We also included four items adapted from NASA Task Load Index (Hart and Staveland, 1988) to assess subjective cognitive cost associated with the empathy decks (e.g., “I worked very hard to accomplish my level of performance in empathy decks”; “The empathy decks were mentally demanding”; α = 0.70). The instrument concluded with basic demographic information.

### Results and Discussion

#### Manipulation Check: Loneliness

A manipulation check was conducted to ensure that the loneliness recall task activated feelings of loneliness. Responses to the loneliness scale before the empathy-selection tasks were averaged to form a loneliness score for each participant. ANOVA results indicated that the manipulation worked as expected. Participants in the lonely condition felt significantly more lonely than those in the control condition (*M*_lonely_ = 3.18, *SD* = 0.40; *M*_control_ = 2.25, *SD* = 0.50), *F*(1, 93) = 100.40, *p* < 0.001, η^2^*_p_* = 0.52.

#### Empathy Choice

Next, we computed the proportion of choosing empathy decks among all trials (*n* = 30) to indicate the tendency to empathize with strangers. For each participant, the value of the empathy choice ranged from 0 to 1. As shown in Table 1, ANOVA results indicated that compared to non-lonely, lonely participants were more likely to choose empathy (*M*_lonely_ = 0.47, *SD* = 0.09; *M*_control_ = 0.34, *SD* = 0.09), *F*(1, 93) = 100.40, *p* < 0.001, η^2^*_p_* = 0.33. The findings are consistent with H_1_.

**TABLE 1:** The mean of empathy choice in Experiments 1 and 2.

Study	Lonely	Control/Connected	*F-*test
Study1	0.47 (0.09)	0.34 (0.09)	*p* < 0.001
Study2			
Positive empathy	0.53 (0.15)	0.37 (0.13)	*p* < 0.001
Negative empathy	0.32 (0.11)	0.38 (0.14)	*p* = 0.089

#### The Mechanism Underlying the Group Difference in Empathy Choice

The hypothesis to be tested was that lonely people engaged in more empathy decks because they perceived higher level of social support instead of thinking these decks were easier.

Participants’ responses to the four items on perceived social support were averaged into a social support index. To test our hypothesis, we first analyzed perceived social support as a function of emotional state. ANOVA results suggested that compared to non-lonely, lonely participants perceived higher social support from the persons in the decks they initiatively chose to empathize (*M*_lonely_ = 5.02, *SD* = 1.08; *M*_control_ = 3.50, *SD* = 1.02), *F*(1, 93) = 49.29, *p* < 0.001, η^2^*_p_* = 0.35.

Similarly, participants’ responses to the four items on cognitive cost were averaged into a cognitive cost index. An analysis of cognitive cost as a function of emotional state was conducted. ANOVA results suggested that there was no significant difference between lonely and non-lonely individuals’ perceived cognitive costs of empathy decks (*M*_lonely_ = 4.67, *SD* = 0.56; *M*_control_ = 4.59, *SD* = 0.93), *F*(1, 93) = 0.27, *p* = 0.61, η^2^*_p_* = 0.003.

We conducted a multiple mediation analysis (Model 4; bootstrapped with 5,000 draws; Hayes, 2012), where emotional state (lonely = 1, non-lonely = -1) was the independent variable, perceived social support and cognitive cost were multiple mediators, and empathy choice was the dependent variable. The analysis results showed that perceived social support positively predicted empathy choice (β = 0.82, *p* < 0.001), whereas cognitive cost negatively predicted empathy choice (β = -0.12, *p* < 0.05). The bootstrapped analysis revealed that the indirect effect of perceived social support was significant [β = 0.48, *SE* = 0.06, 95% CI (0.37, 0.60) excluded zero], but the indirect effect of cognitive cost was insignificant [β = -0.01, *SE* = 0.02, 95% CI (-0.05, 0.02) included zero]. The results were consistent with H_2_, which suggested that although empathy entails the same level of cognitive cost under the condition of loneliness and control, but lonely participants proactively chose empathy because of perceived social support when they were given right to choose freely.

#### The Beneficial Effect of Empathy on Loneliness Intervention

Next, we examined whether the loneliness score changed before and after completing the empathy-selection task. Responses to the loneliness scale completed after the empathy-selection task were averaged to form a post-task loneliness score for each participant. Loneliness scores before and after the empathy-selection task in two emotional states were submitted to a mixed-measures ANOVA. Results indicated a significant main effect of the time node, *F*(1, 93) = 87.84, *p* < 0.001, η^2^*_p_* = 0.49. More importantly, this main effect was qualified by a significant interaction between time node and emotional state, *F*(1, 93) = 58.13, *p* < 0.001, η^2^*_p_* = 0.39. Specifically, there was a significant difference in the loneliness scores between the lonely and control condition before the empathy-selection task (*M*_lonely_ = 3.18, *SD* = 0.40; *M*_control_ = 2.25, *SD* = 0.50), *F*(1, 93) = 100.40, *p* < 0.001, η^2^*_p_* = 0.52. However, after the empathy-selection task, no significant difference was detected between these two conditions (*M*_lonely_ = 2.02, *SD* = 0.53; *M*_control_ = 2.14, *SD* = 0.68), *F*(1, 93) = 0.86, *p* = 0.36, η^2^*_p_* = 0.01. These results confirmed our prediction that empathy choice allowed lonely participants to decrease loneliness.

Furthermore, to examine the important role of perceived social support and empathy choice in the loneliness intervention, we ran a serial mediation analysis with emotional state as the independent variable (lonely = 1, control = -1), decrease in loneliness (calculated by pre-task minus post-task loneliness) as the dependent variable, and perceived social support and empathy choice as mediators. In accordance with the development of serial mediation macros presented by Preacher and Hayes (2008), the standard value of the direct and indirect coefficients in the relationship between emotional state and decrease in loneliness were calculated. All the path coefficients stand for regression weights in the relationship between independent and dependent variables.

**FIGURE 1:**
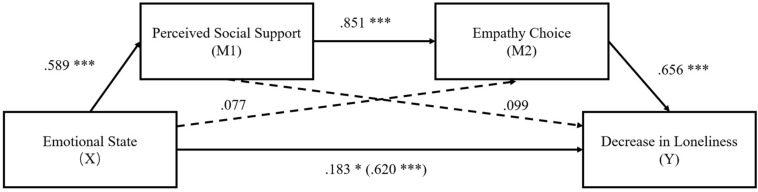
The multiple serial mediation model in Experiment 1. Emotional state (Lonely = 1; Control = −1). All the path coefficients are standardized.

As shown in Figure 1, the total effect (β = 0.62, *p* < 0.001) from emotional state to decrease in loneliness was at a significant level (Step 1). Moreover, the direct path from emotional state to perceived social support (β = 0.59, *p* < 0.001) was significant. Meanwhile, the path from the first mediator (perceived social support) to the second mediator (empathy choice) was also significant (β = 0.85, *p* < 0.001) (Step 2). The path from the second mediator (empathy choice) to decrease in loneliness was significant (β = 0.66, *p* < 0.001) (Step 3). However, the direct path from emotional state to decrease in loneliness became weaker (β = 0.18, *p* < 0.05) (Step 4). Table 2 shows the indirect effects and their associated 95% CIs. As shown in the Table, the total indirect effect of emotional state through perceived social support and empathy choice was significant (β = 0.44, *p* < 0.001). The serial mediation of perceived social support and empathy choice was significant (β = 0.33, *p* < 0.001), whereas the single mediation of perceived social support (β = 0.06, *p* = 43) and empathy choice (β = 0.05, *p* = 0.18) were not significant. These results are consistent with H_3_.

**TABLE 2:** Bootstrapping indirect effects and 95% confidence interval (CI) for the multiple serial mediation model in Experiment 1.

Number	Model pathways	Point estimate β	95%CI	
			
			Lower	Upper
1	Total indirect effect	0.437	0.332	0.551
2	Emotional state → Perceived social support → Decrease in loneliness	0.058	−0.085	0.206
3	Emotional state → Empathy choice → Decrease in loneliness	0.050	−0.020	0.129
4	Emotional state → Perceived social support → Empathy choice → Decrease in loneliness	0.329	0.212	0.475

Results suggested that the higher level of social support embedded with empathy decks, as compared to description decks, is the motivator for lonely (vs. non-lonely) people to increase their tendency to embrace empathy, which serves as an adapted strategy for them to effectively reduce their loneliness.

## Experiment 2

The results of Experiment 1 support our hypothesis that lonely (vs. non-lonely) participants are more willing to empathize with someone who experiences positive states, which increases perceived social support and in turn decreases their perceived loneliness. However, hypervigilance to social information depends on the emotional valence of expressions (Bangee et al., 2014). Experiment 2 was conducted to investigate the interaction effect of the valence of empathy on the manipulation of loneliness on the empathy engagement and specifically examine what would happen when the empathy decks were embedded with images of negative emotions. Research (e.g., DeWall et al., 2009; Saito et al., 2020) provided evidence that the negative experience of loneliness and the threat of social exclusion increased selective attention to signs of social acceptance (smiling faces) than to that conveying social disapproval (sad/competent faces). Our hypothesis to be tested in Experiment 2 was that lonely people would show a higher tendency to avoid negative empathy than non-lonely participants, as the effect of loneliness on empathy engagement is based on perceived social support. The experiment employed a 2 (emotional state: lonely vs. connected) by 2 (empathy valence: positive vs. negative) between-subjects design, where loneliness was manipulated by the similar procedure of Experiment 1 and empathy valence was manipulated by varying the target affect.

### Methods

#### Participants

One hundred and fifty-five college students (63 males, 92 females, *M*_age_ = 19.82 years, *SD*_age_ = 1.97) participated and received financial rewards. None of them have participated in Experiment 1. As prosocial behavior can function as a signal of an individual’s resources or good character (Smith and Bird, 2000), some prosocial behaviors are motivated by a desire to acquire reputation (Milinski et al., 2002), trust (Barclay, 2005), and other social and psychological objectives (Omoto and Snyder, 1995), especially in a situation with public feedback (Böhm and Regner, 2013). To rule out the possibility that participants’ prosocial tendency to empathize was motivated by social desirability, we informed participants that they were making contributions for a face database anonymously, and none of their personal information would be made public.

### Design and Procedure

A similar procedure in Experiment 1 to activate feelings of loneliness was adopted. Participants were instructed to recall an emotional experience. In the lonely condition, participants were asked to write about a time they felt lonely and isolated. In the connected condition, participants were asked to write a time they felt companionship and connectedness. Then they completed the pre-task UCLA loneliness subscale (α = 0.91). The design of the empathy-selection task was identical to Experiment 1, except that empathy valence was manipulated between subjects. Target images were two types of emotions of 15 male and female adults from the CAS-PEAL Large-Scale Chinese Face Database (Gao et al., 2008). In the positive condition, each face model was smiling, displaying joy or happiness; in the negative condition, each face model was frowning, displaying worry or anger (Supplementary Presentation S1, S2). Finally, they completed the same post-task loneliness subscale (α = 0.87) and assessments of the empathy decks [including perceived social support (α = 0.85) and cognitive cost (α = 0.89)] as in Experiment 1].

### Results and Discussion

#### Manipulation Check: Loneliness

Responses to the loneliness scale before the empathy-selection tasks were averaged to form a loneliness score for each participant. ANOVA results proved the loneliness manipulation caused specific increase in subjective ratings of loneliness. Participants in the lonely condition felt significantly more lonely than those in the connected condition (*M*_lonely_ = 3.11, *SD* = 0.59; *M*_connected_ = 2.10, *SD* = 0.56), *F*(1, 153) = 118.95, *p* < 0.001, η^2^*_p_* = 0.44.

#### Empathy Choice

A 2 (emotional state) × 2 (empathy valence) ANOVA indicated a significant main effect of emotional state, such that lonely participants chose more empathy decks than non-lonely participants, *F*(1, 151) = 5.24, *p* < 0.05, η^2^*_p_* = 0.03. More importantly, this main effect was qualified by a significant interaction between emotional state and empathy valence, *F*(1, 151) = 22.26, *p* < 0.001, η^2^*_p_* = 0.13. Specifically, simple effect in the positive empathy condition revealed that, compared to non-lonely participants (*M_connected_* = 0.32, *SD* = 0.13), lonely participants were more likely to engage in more empathy decks (*M_lonely_* = 0.53, *SD* = 0.15), *F*(1, 151) = 24.70, *p* < 0.001, η^2^*_p_* = 0.14. However, lonely participants were more likely to avoid negative empathy trials (*M_lonely_* = 0.32, *SD* = 0.11) than non-lonely participants (*M_connected_* = 0.38, *SD* = 0.14), *F*(1, 151) = 2.93, *p* = 0.09, η^2^*_p_* = 0.02.

#### The Mechanism for the Interaction Effect of Emotional State and Empathy Valence on Empathy Choice

An analysis of perceived social support as a function of emotional state and empathy valence yielded a significant two-way interaction, *F*(1, 151) = 22.01, *p* < 0.001, η^2^*_p_* = 0.13. Simple effects confirmed that, compared to non-lonely, lonely participants indicated higher perceived social support from positive empathy decks (*M*_lonely_ = 4.66, *SD* = 1.16; *M*_connected_ = 3.47, *SD* = 1.16), *F*(1, 151) = 24.17, *p* < 0.001, η^2^*_p_* = 0.14. Conversely, in the negative empathy condition, lonely participants perceived lower social support than non-lonely participants (*M*_lonely_ = 3.50, *SD* = 1.07; *M*_connected_ = 3.92, *SD* = 0.88), *F*(1, 151) = 2.99, *p* = 0.086, η^2^*_p_* = 0.02.

Similarly, an analysis of cognitive cost as a function of emotional state and empathy valence found no significant main effects of emotional state [*F*(1, 151) = 0.20, *p* = 0.66, η^2^*_p_* = 0.001] or empathy valence [*F*(1, 151) = 0.13, *p* = 0.72, η^2^*_p_* = 0.001]. The two-way interaction of emotional state and empathy valence was insignificant either, *F*(1, 148) = 0.24, *p* = 0.63, η^2^*_p_* = 0.002. Results showed that, much like non-lonely participants, lonely participants considered the empathy decks as equally mentally demanding (*M*_lonely_ = 4.24, *SD* = 1.09; *M*_connected_ = 4.16, *SD* = 1.30), which is consistent with the findings in Experiment 1. Besides, the positive and the negative empathy decks consumed the same amount of cognitive resources (*M*_positive_ = 4.17, *SD* = 1.23; *M*_negative_ = 4.24, *SD* = 1.17).

We conducted a follow-up mediated moderation analysis (Hayes, 2012; model 8; bootstrapped with 5,000 draws), and the emotional state (lonely = 1, connected = -1) was entered as the independent variable, empathy valence (positive = 1; negative = -1) as the moderator, perceived social support and cognitive cost as the multiple mediators, and empathy choice as the dependent variable. As predicted, perceived social support [β = 0.16, *SE* = 0.04, 95% CI (0.10, 0.23) excluded zero], but not cognitive cost (β = 0.01, *SE* = 0.02, 95% CI (−0.02, 0.05) included zero] mediated the interaction effect of emotional state and empathy valence on empathy choice. Specifically, perceived social support mediated the increase in empathy choice for lonely participants relative to non-lonely participants in the positive empathy condition [β = 0.26, *SE* = 0.07, 95% CI (0.15, 0.41) excluded zero], replicating the pattern demonstrated in Experiment 1. Conversely, the reverse pattern was found in negative empathy condition, such that perceived social support mediated the decrease in empathy choice for lonely participants relative to non-lonely participants in the negative empathy condition [β = −0.08, *SE* = 0.04, 95% CI (−0.17, −0.004) excluded zero].

#### The Moderation Effect of Empathy Valence on Loneliness Intervention

**FIGURE 2:**
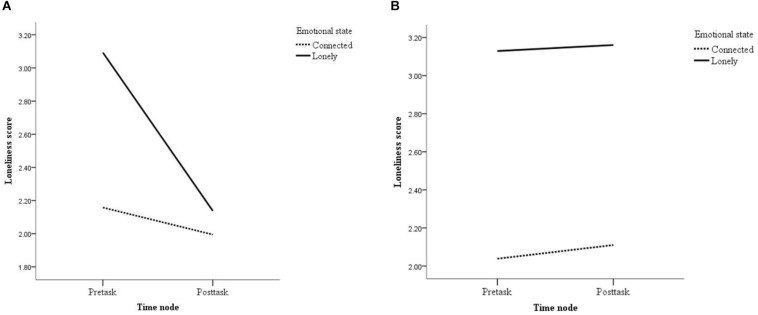
Loneliness score in Experiment 2. **(A,B)** Respectively depict the interaction effect of emotional state and time node on loneliness score under the condition of positive empathy and under the condition of negative empathy.

An analysis of loneliness score was submitted to a 2 (emotional state: lonely vs. connected) × 2 (empathy valence: positive vs. negative) × 2 (time node: pre-task vs. post-task) mixed-measures ANOVA. Results showed that the main effect of time node was significant, *F*(1, 151) = 18.20, *p* < 0.001, η^2^*_p_* = 0.11. More importantly, a significant three-way interaction was detected, *F*(1, 151) = 9.98, *p* = 0.001, η^2^*_p_* = 0.06, such that the variability in loneliness score before and after the empathy-selection task was affected by the interaction of emotional state and empathy valence. Specifically, as shown in Figure 2, under the condition of positive empathy, participants in the lonely condition were more lonely than those in the connected condition before completing the empathy-selection task (*M*_lonely_ = 3.09, *SD* = 0.61; *M*_connected_ = 2.16, *SD* = 0.49), *F*(1, 151) = 50.57, *p* < 0.001, η^2^*_p_* = 0.25; whereas no significant group difference was ever found after the empathy-selection task (*M*_lonely_ = 2.14, *SD* = 0.48; *M*_connected_ = 1.99, *SD* = 0.49), *F*(1, 151) = 1.67, *p* = 0.20, η^2^*_p_* = 0.01. However, under the condition of negative empathy, the effect of the emotional state on loneliness score was still significant and even stronger after the task (*M*_lonely_ = 3.13, *SD* = 0.58; *M*_connected_ = 2.04, *SD* = 0.63), *F*(1, 151) = 89.20, *p* < 0.001, η^2^*_p_* = 0.37, as compared to the effect before the task, (*M*_lonely_ = 3.16, *SD* = 0.51; *M*_connected_ = 2.11, *SD* = 0.47), *F*(1, 151) = 67.99, *p* < 0.001, η^2^*_p_* = 0.31. The results suggested that proactively engaging oneself in positive empathy would help lonely people to decrease their loneliness effectively, whereas negative empathy fails to play a part in the loneliness intervention.

To examine the important role of perceived social support and empathy engagement in mediating the interaction effect of emotional state and empathy valence on loneliness intervention, we ran a follow-up serial mediation analysis (Model 6; bootstrapped with 5,000 draws; Hayes, 2012) with the decrease in loneliness (calculated by pre-task minus post-task loneliness score) as the dependent variable and the emotional state (lonely = 1, connected = -1), emotional valence (positive = 1, negative = −1), and their two-way interaction as the independent variables. The total effect (β = 0.22, *p* = 0.001) from the two-way interaction to decrease in loneliness was at a significant level (Step 1). Moreover, the direct path from the interaction to the first mediator (perceived social support) was significant (β = 0.35, *p* < 0.001). Meanwhile, the path from the first mediator (perceived social support) to the second mediator (empathy choice) was also significant (β = 0.46, *p* < 0.001) (Step 2). The path from the second mediator (empathy choice) to decrease in loneliness were significant (β = 0.29, *p* = 0.002) (Step 3). However, the direct path from emotional state to decrease in loneliness became insignificant (β = 0.05, *p* = 0.48) (Step 4). Furthermore, bootstrap analyses revealed that the total indirect effect of the interaction of emotional state and empathy valence through perceived social support and empathy choice was significant [β = 0.18, *p* < 0.001, 95% CI (0.11, 0.26) excluded zero]. Specifically, the single mediation of social support (M_1_) [β = 0.08, *p* < 0.05, 95% CI (0.02, 0.16) excluded zero], the multiple serial mediations of social support and empathy choice [β = 0.05, *p* < 0.05, 95% CI (0.02, 0.10) excluded zero], and the single mediation of empathy choice (M_2_) [β = 0.05, *p* < 0.05, 95% CI (0.01, 0.11) excluded zero] in the interaction effect of emotional state and empathy valence on decrease in loneliness score were all significant in the tested model.

Findings of Experiment 2 confirmed our prediction that lonely individuals attach great importance to social support, a factor which powerfully shapes their empathy choice and determines the degree of the decline in perceived loneliness. It was demonstrated that compared to non-lonely people, lonely people are more willing to empathize with positive affect and effectively reduce their loneliness as a result, whereas they will run away from negative emotional expressions because of their perceived risk of decreasing social support.

## General Discussion

Our research has been the first to investigate how subjective loneliness would affect empathy engagement. Rather than simply asking people to self-report their empathy, we adopted an empathy selection paradigm to observe how lonely and non-lonely people choose between situations and tested the mechanism underlying the phenomenon.

Other studies have shown that subjective loneliness have effects on automatic procession of social information, including enhanced sensitivity to emotional tone (Pickett et al., 2004) and heightened visual attention to facial information conveying social acceptance (Saito et al., 2020). The present study advances the research of loneliness by examining its effect on behavioral empathy choice. Results showed that when given the opportunity to share in others’ experiences, compared to non-lonely people, lonely people automatically preferred to empathize more with strangers in positive emotional states but got involved in fewer negative empathy decks.

Furthermore, the mediated moderation analysis supported our hypothesis that perceived social support was shown to be a significant mediator between the relationship of loneliness and empathy engagement, indicating that compared to non-lonely, lonely people perceived much more social support from positive empathy and less from negative empathy. Although numerous studies consistently found that empathy can be overly costly, leading to fatigue, financial costs, and opportunity costs (Zaki, 2014), the findings of the present study support the prediction that empathy would vary as a function of the social environment, and perceived loneliness created a situation which scaffolds empathy with social reward, and this is in line with findings that prosocial behaviors can lead to increased positive feelings and hedonic benefits (for reviews, see Aknin et al., 2018). Specifically, perceived social support served to be a powerful factor to shape empathy in lonely condition.

Delving deeper, we found that our results were consistent with the finding that loneliness is a motivational factor for building and maintaining social connections (Vanhalst et al., 2012). Generally speaking, there were two competing motives for prosocial behaviors, including egoistic motivation and pure altruism (Batson et al., 1989). Note that empathy helps to provide social support for lonely people, it can be speculated that most of prosocial behaviors, regardless how noble in appearance, are motivated by some form of self-benefits. However, it was also indicated in our research that empathy serves as an adaptive tool in the regulatory system, which will be activated by loneliness to monitor social information that may provide cues to inclusion and belonging.

Our results also point to the potential interventions for decreasing loneliness by manipulating their social cognition. An integrative meta-analysis of loneliness intervention (Masi et al., 2011) revealed that correcting maladaptive social cognition was the most helpful way to reduce loneliness compared to improving social skills, enhancing social support by group interventions, and increasing opportunities for social contact, which shed light on the development of cognitively oriented programs. It is worth noting that basically aiming at increasing opportunities for social contact is a deviation from the reality that loneliness may be a result of lacking time or social skills to develop ideal relationships. Although physical social interactions are not replaceable, it would be an encouraging news that empathy would rather be an alternative approach for social contact opportunities and serve as a self-helping intervention tool.

Future research should explore how empathy choice varies as a function of different forms of loneliness. A limitation in the present research is that loneliness is manipulated by a recall task, and we did not classify it as a chronic trait or a transient feeling. Because loneliness can be experienced provisionally in response to an experience of social rejection or to a shift in circumstances (e.g., starting college, divorce, or relocating to a new city), and it can also be experienced more chronically, functioning as a trait-like characteristic (Cacioppo and Patrick, 2008), future studies are needed to investigate whether loneliness, as a trait or as a state, would affect empathy differently. From another perspective, loneliness is a complex set of feelings encompassing reactions to denial of intimate and social needs. One theory of loneliness posits that dissatisfaction with specific types of relationships (e.g., social network members vs. romantic partner) leads to different types of loneliness (e.g., social vs. emotional) (Weiss, 1973). Thus, further studies are needed to clarify the different effect of social and emotional loneliness on empathy choice.

Moreover, our research represents an important launching point for exploring the moderation role of empathy valence in the loneliness intervention effect. However, how loneliness intervention varies as a function of specific types of emotion and the intensity of emotion is worth deeper investigation. Specifically, the present research revealed that lonely people perceived less social support from negative emotional expressions. The face models used in the negative empathy condition (Experiment 2) were frowning, a prototypical facial expression of anger or worry (Ekman and Friesen, 1975); it is therefore unclear whether the effect of negative facial expression on loneliness intervention would be dependent on specific types of negative emotions. For example, it is important to explore whether lonely people would respond differently toward anger (a particular negative emotion expressed outwardly) or worry (expressed relatively internally). Besides, another worthwhile avenue to explore is the moderation role of emotion intensity. Exploring these ideas would provide important boundary conditions for the effect of empathy engagement on loneliness intervention.

## Conclusion

Our research advances the study of loneliness by suggesting that subjective loneliness creates a situation that scaffolds empathy with social reward. Compared to non-lonely people, lonely people act more prosocially and empathize more with emotional faces. This pattern occurs only for positive empathy because they perceive higher social support from sharing in others’ positive emotions. However, lonely people tend to avoid negative empathy because of decreased social support. Furthermore, the current work contributes to the broader discussion about motivated empathy, which suggests that although empathy do entail cognitive cost, but empathy choice would fluctuate across different social environments in which these decisions are being made.

## Data Availability Statement

The datasets generated for this study are available on request to the corresponding author.

## Ethics Statement

The studies involving human participants were reviewed and approved by Ethics Committee of the Department of Psychology, Sun Yat-sen University. Written informed consent to participate in this study was provided by the participants and their’ legal guardian/next of kin where required.

## Author Contributions

TH conceived of the idea with the assistance of XZ and MH. TH collected and analyzed all data for two experiments with the assistance of XZ. TH and XZ wrote and revised the manuscript. MH provided consultation for experiment design and provided feedback to the manuscript. All authors approved the final manuscript.

## Conflict of Interest

The authors declare that the research was conducted in the absence of any commercial or financial relationships that could be construed as a potential conflict of interest.

## Supplementary Material

PRESENTATION S1 The face models used in the present research smiling face models.

PRESENTATION S2 The face models used in the present research frowning face models.
