# Supplementary material for: Absence and Presence of Human Interaction: The Relationship Between Loneliness and Empathy
Source: Front Psychol. 2020 May 19;11:768. doi: 10.3389/fpsyg.2020.00768 (PMC7249960; doi:10.3389/fpsyg.2020.00768)

## Slide 1
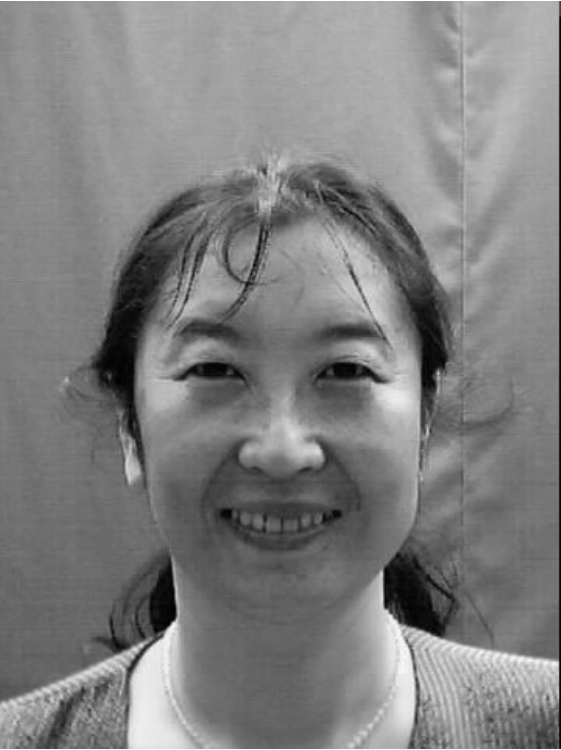

## Slide 2
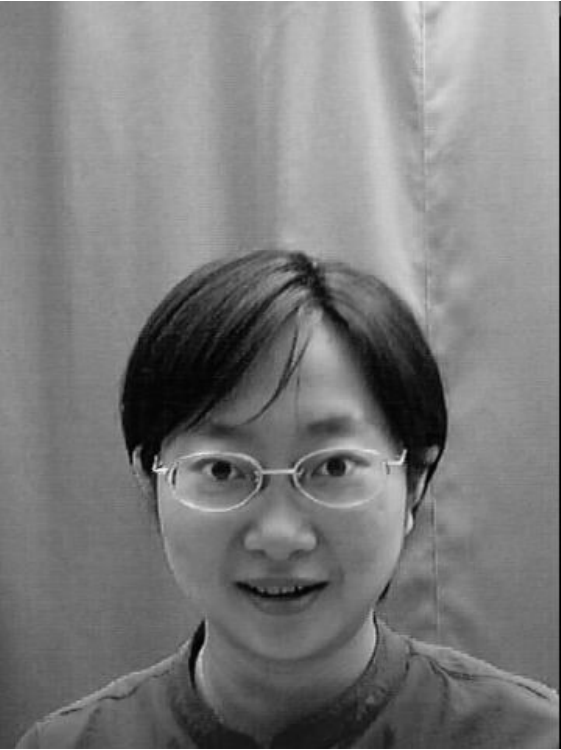

## Slide 3
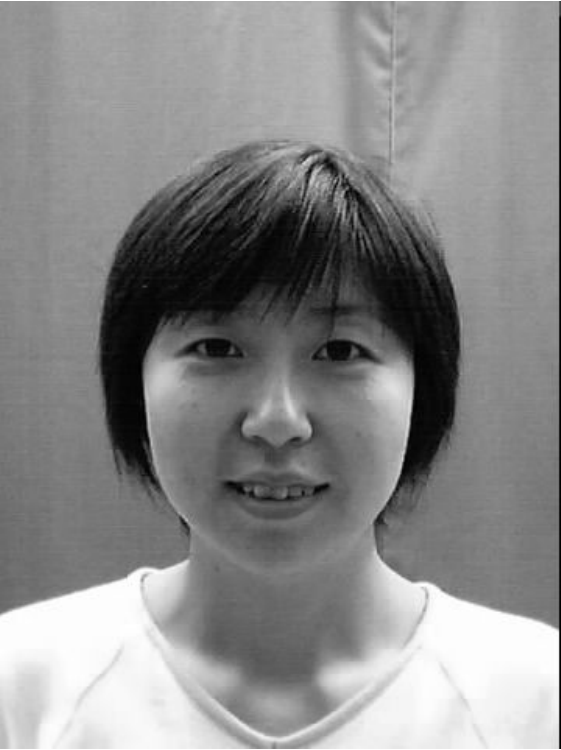

## Slide 4
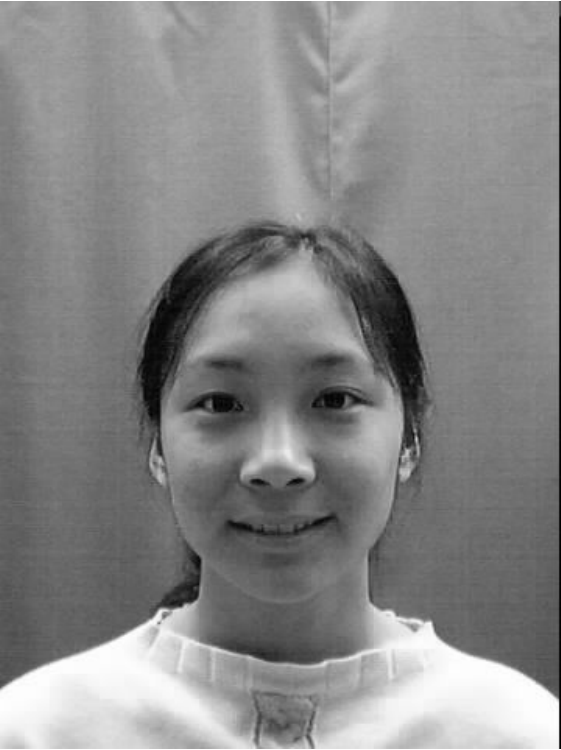

## Slide 5
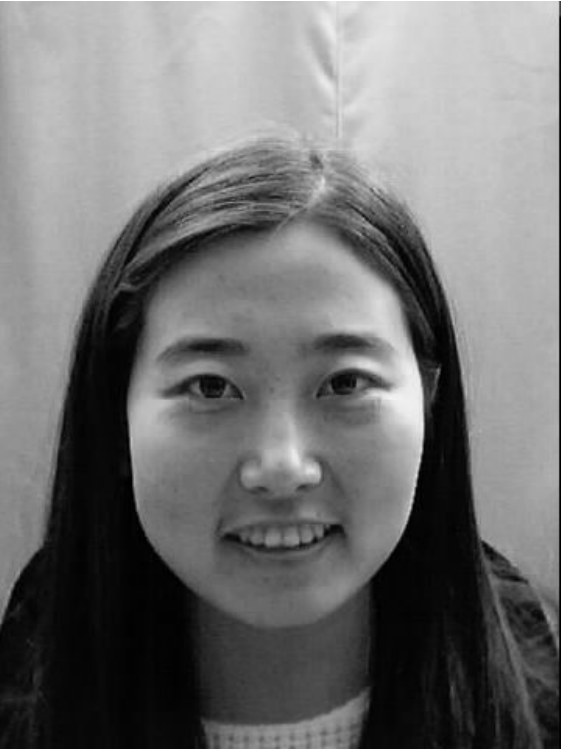

## Slide 6
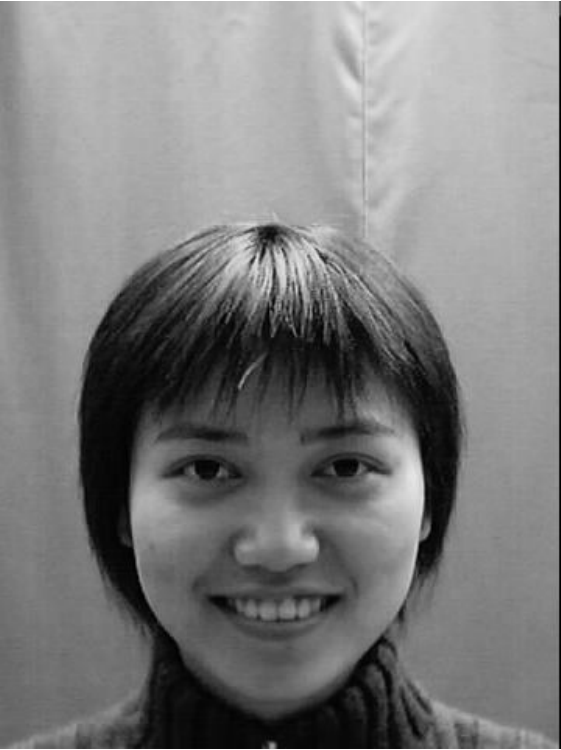

## Slide 7
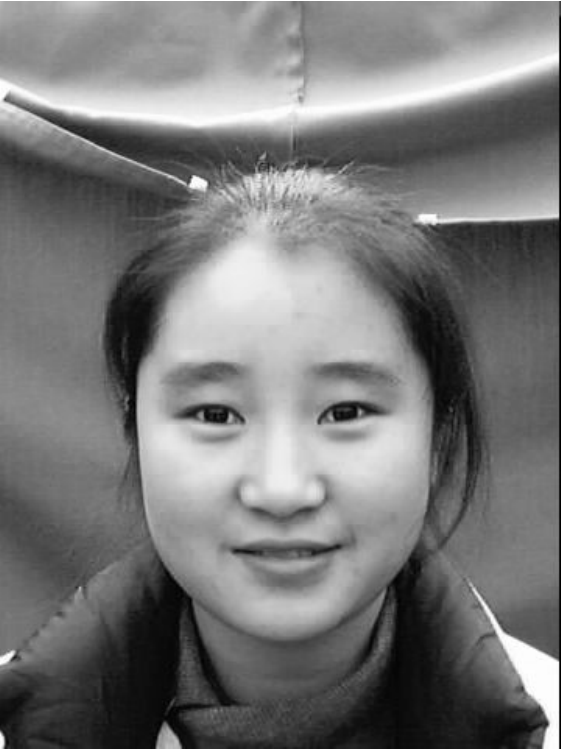

## Slide 8
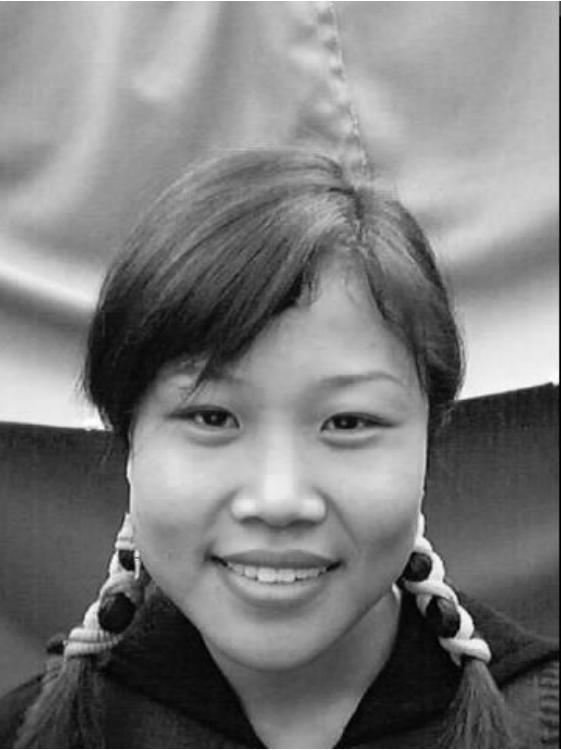

## Slide 9
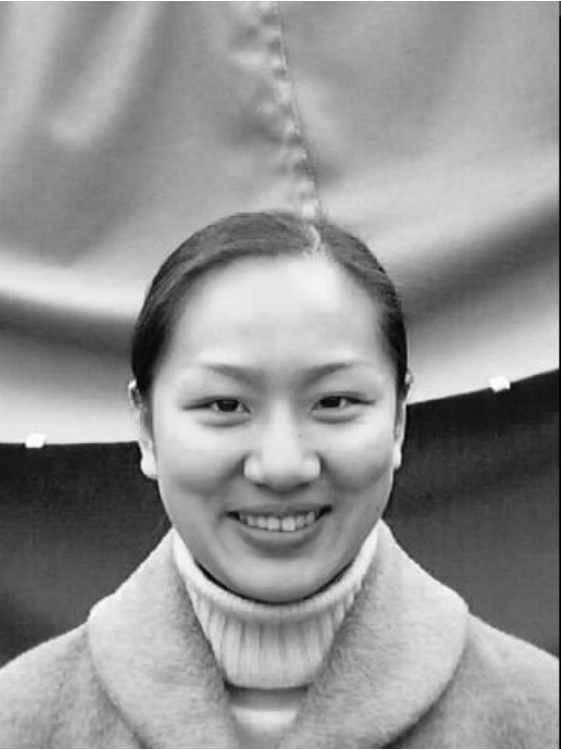

## Slide 10
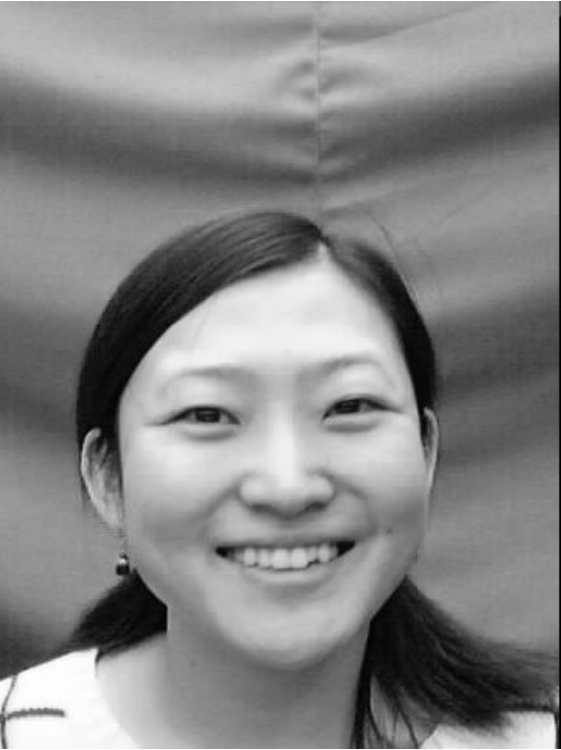

## Slide 11
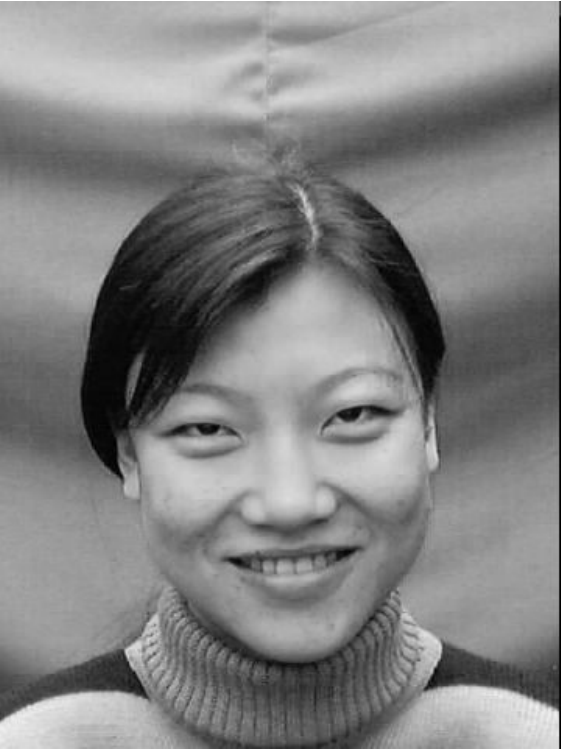

## Slide 12
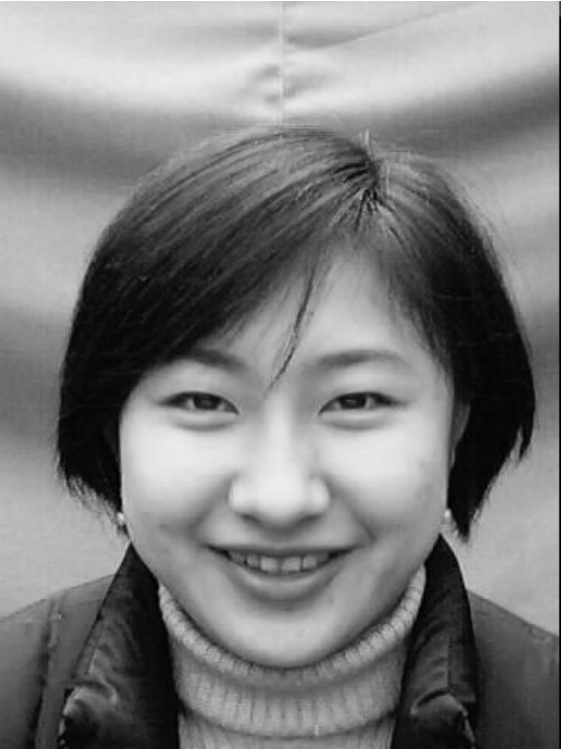

## Slide 13
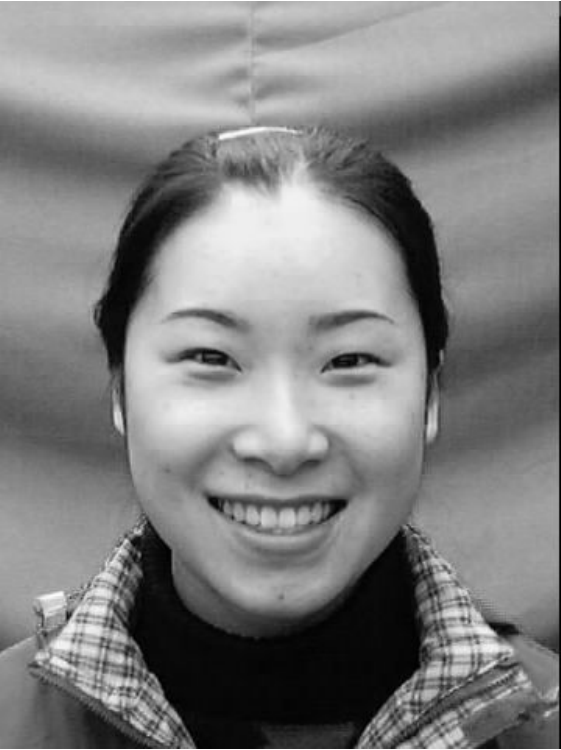

## Slide 14
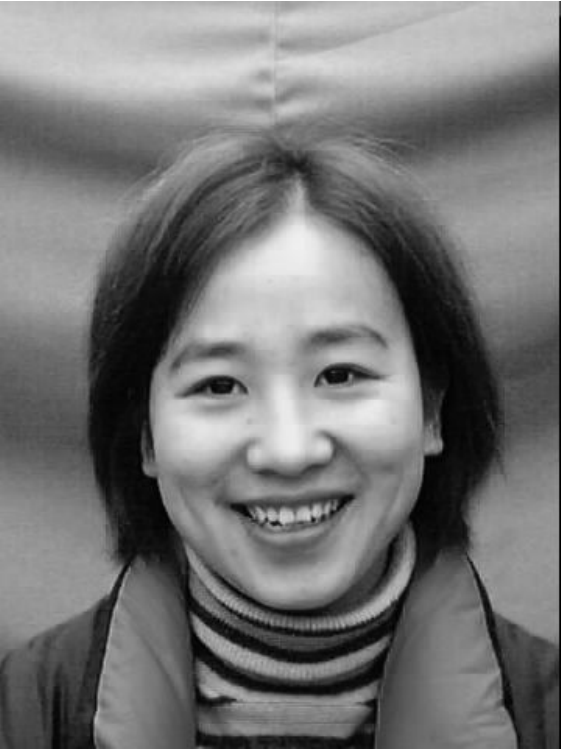

## Slide 15
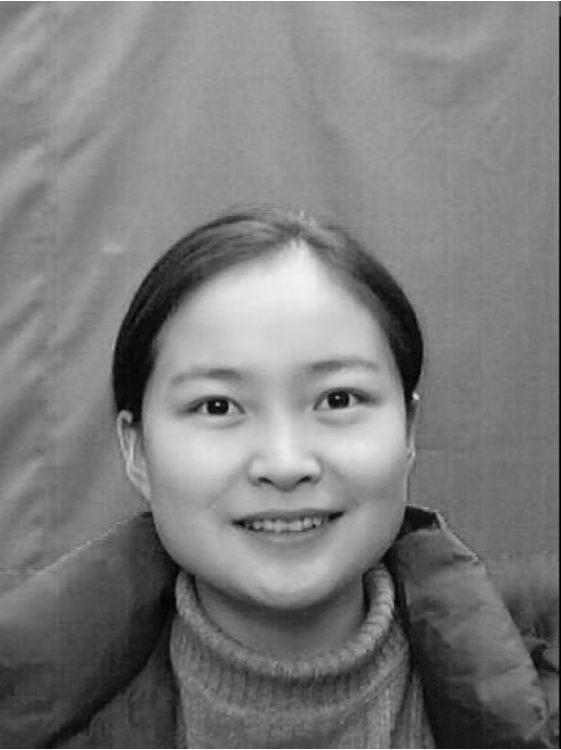

## Slide 16
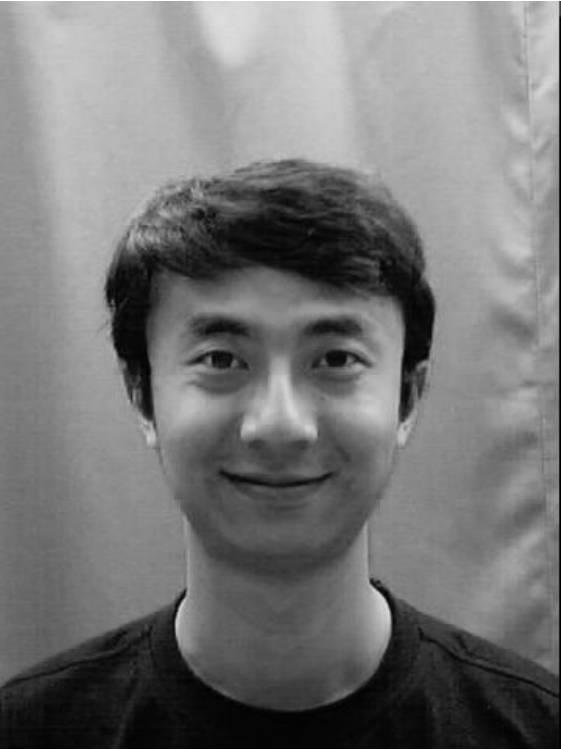

## Slide 17
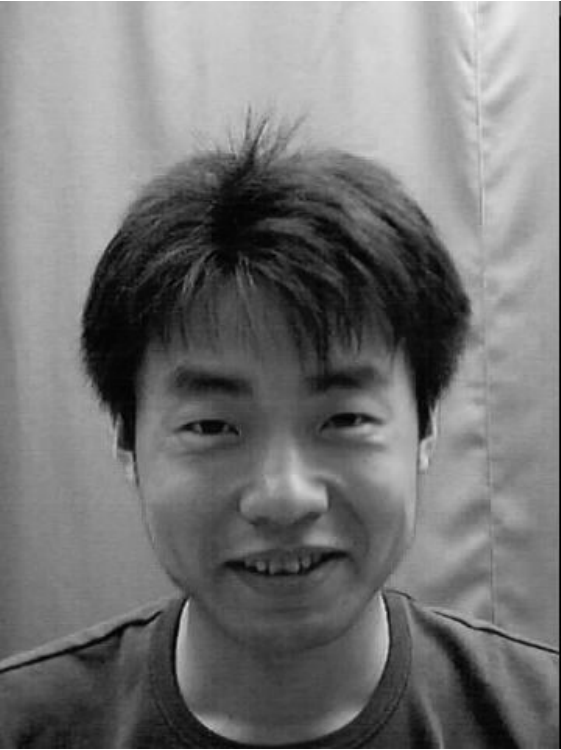

## Slide 18
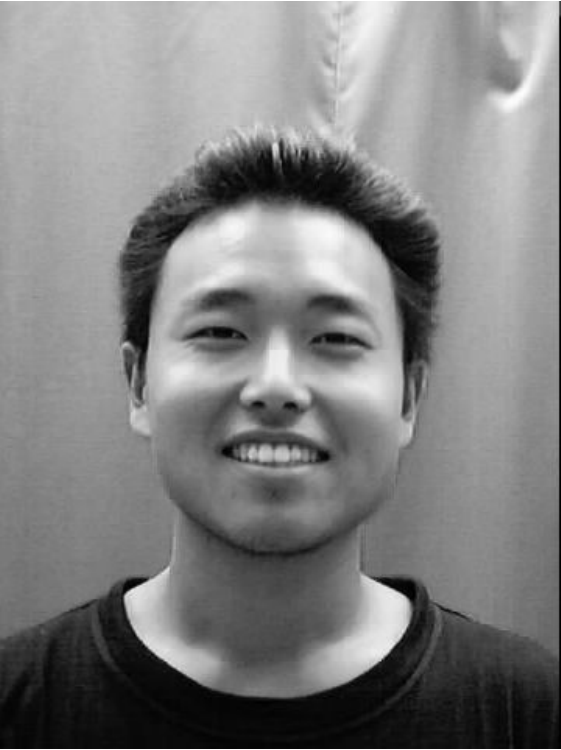

## Slide 19
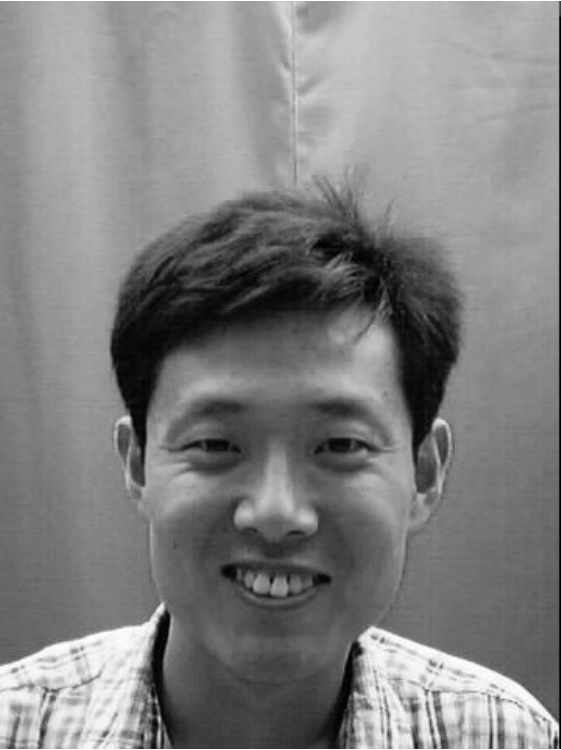

## Slide 20
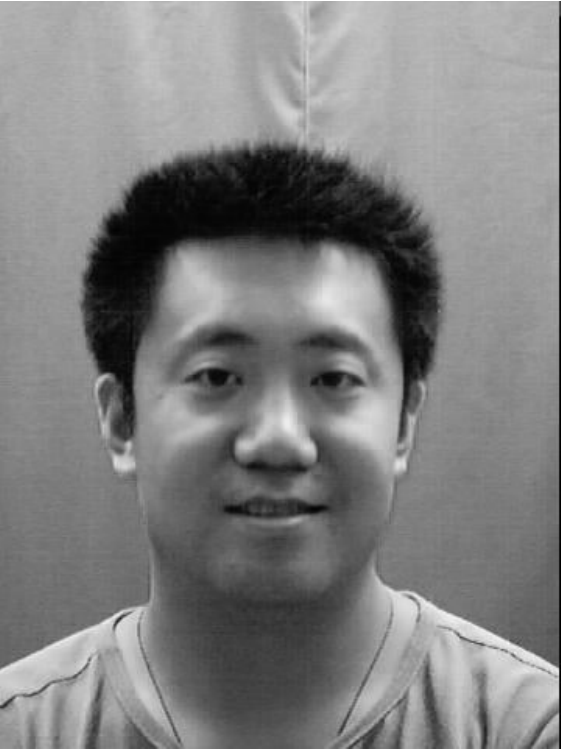

## Slide 21
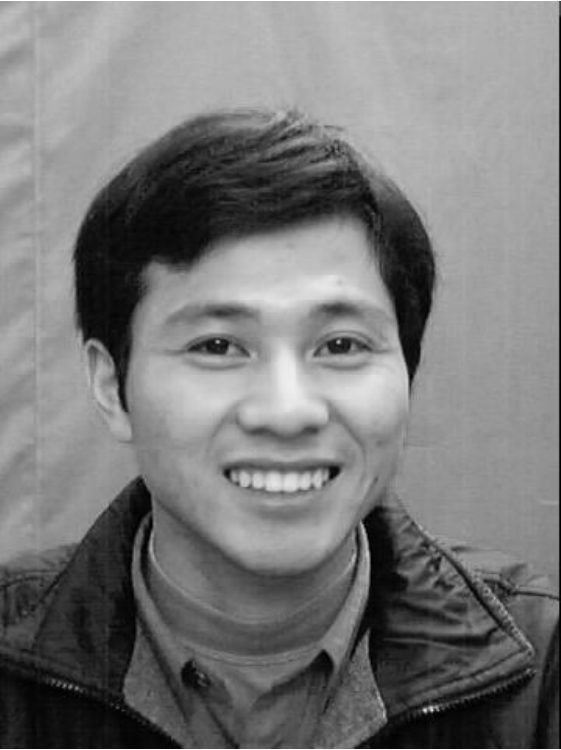

## Slide 22
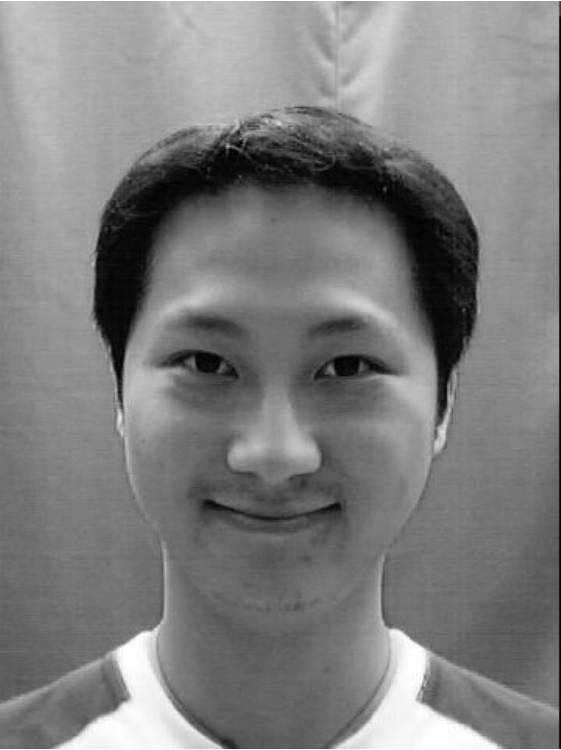

## Slide 23
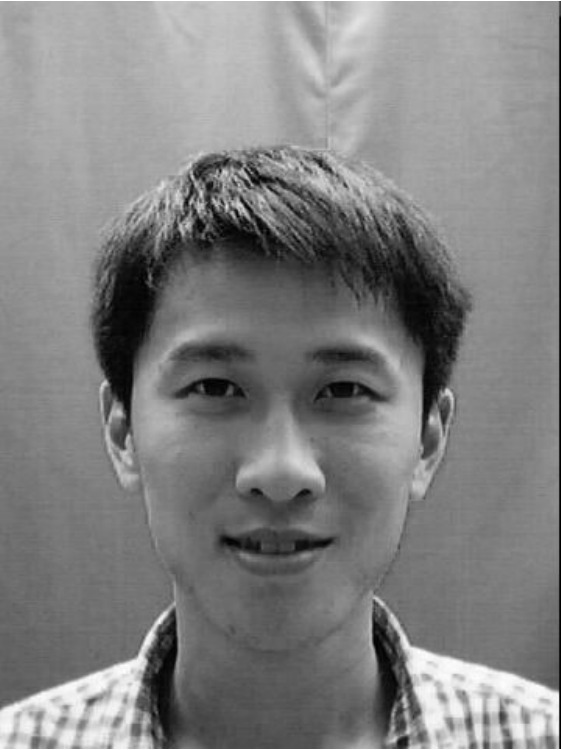

## Slide 24
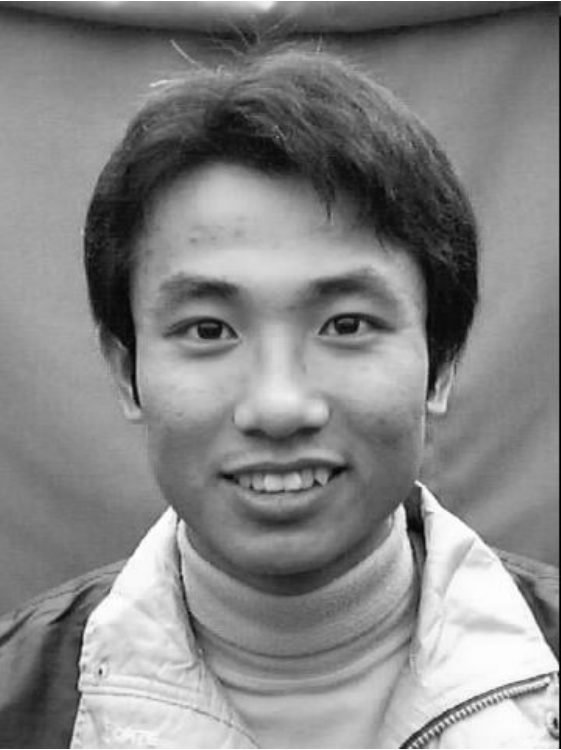

## Slide 25
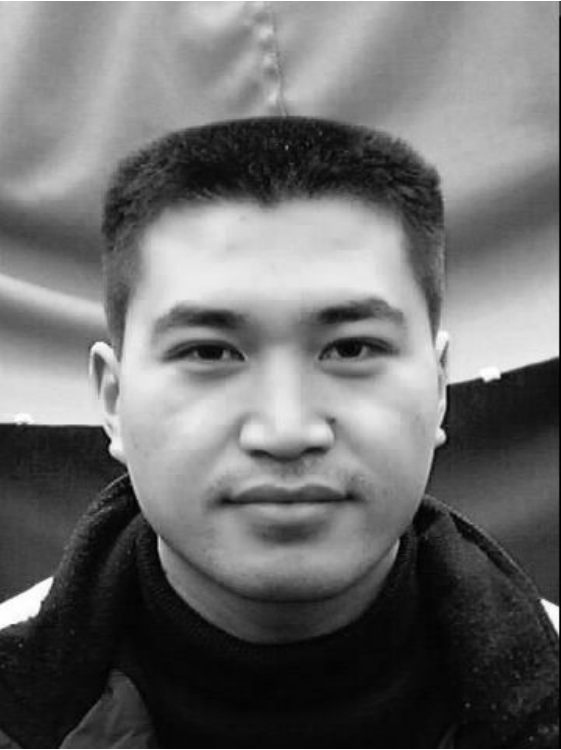

## Slide 26
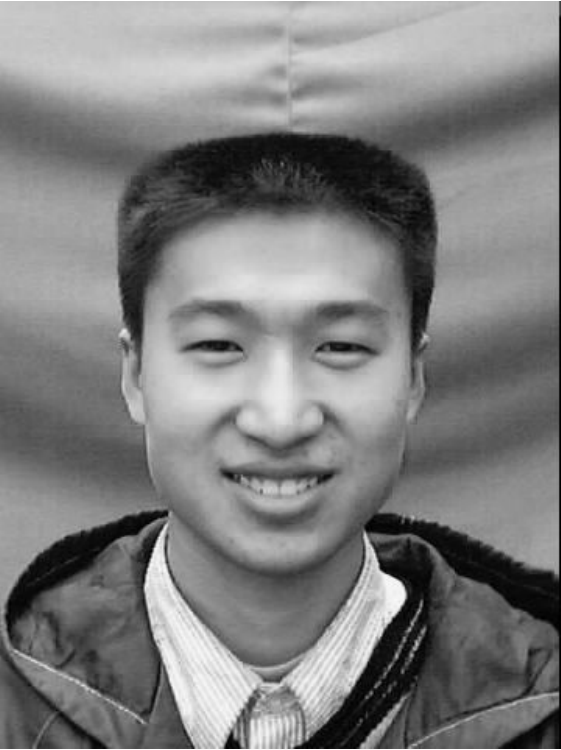

## Slide 27
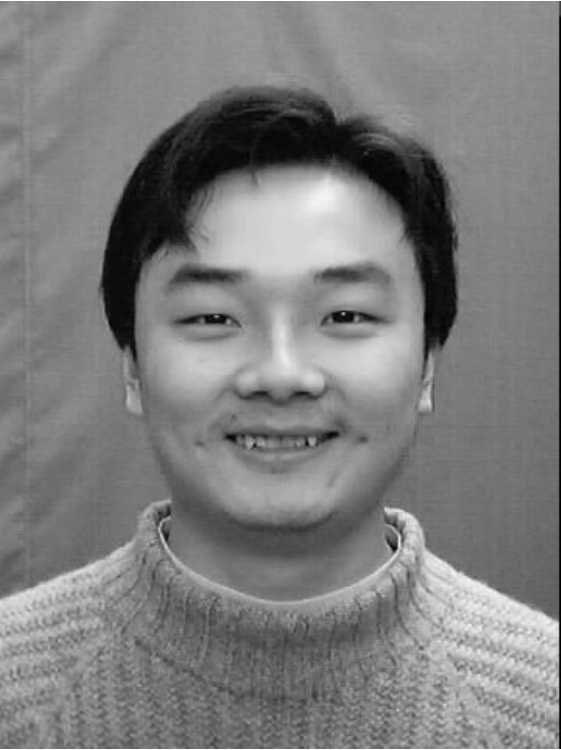

## Slide 28
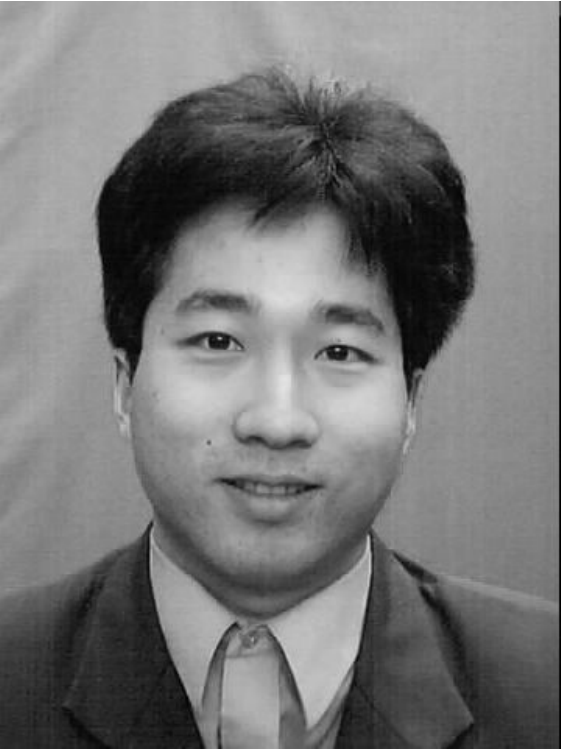

## Slide 29
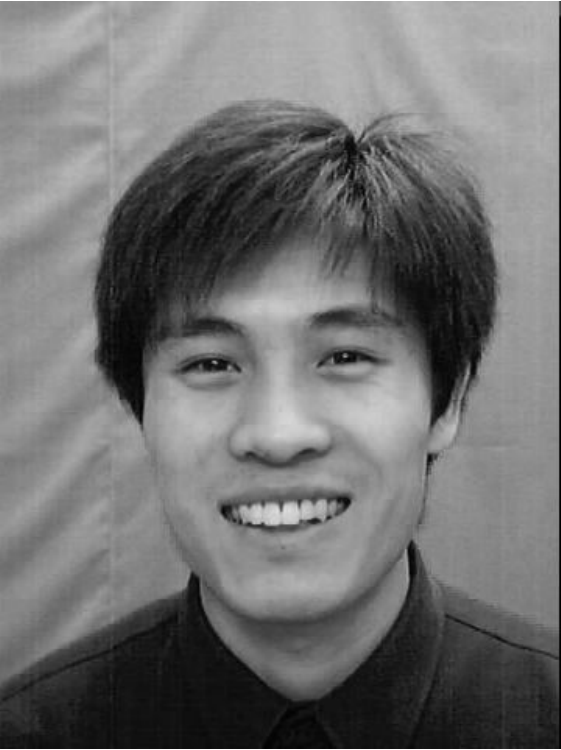

## Slide 30
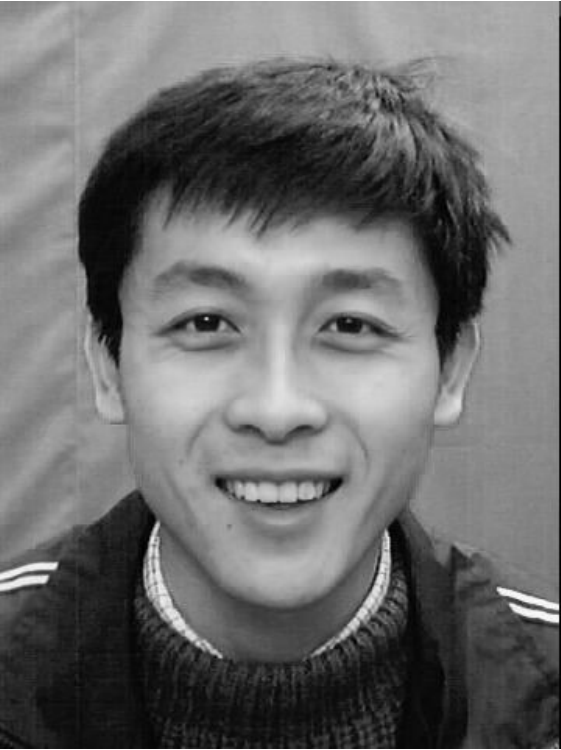

Supplement: PRESENTATION S1 — The face models used in the present research smiling face models. [file Presentation_1.pptx]
